# Construction and Validation of Cardiovascular Disease Prediction Model for Dietary Macronutrients—Data from the China Health and Nutrition Survey

**DOI:** 10.3390/nu16234180

**Published:** 2024-12-02

**Authors:** Jia Guo, Yanyan Dai, Yating Peng, Liangchuan Zhang, Hong Jia

**Affiliations:** 1 School of Public Health, Southwest Medical University, Luzhou 646000, China; gjnzb324@outlook.com (J.G.); pyt19990916@hotmail.com (Y.P.); zlc7751801@gamil.com (L.Z.); 2 School of Public Health, Shanxi Medical University, Taiyuan 030001, China; taoyayahu@outlook.com; 3 Collaborating Center of the National Institute of Health Data Sciences of China, Southwest Medical University, Luzhou 646000, China

**Keywords:** cardiovascular disease, dietary macronutrients, predictive model, LASSO, nomogram

## Abstract

Background: There are currently many studies on predictive models for cardiovascular disease (CVD) that do not use dietary macronutrients for prediction. This study aims to provide a non-invasive model incorporating dietary information to predict the risk of CVD in adults. Methods: The data for this study were obtained from the China Health and Nutrition Survey (CHNS) spanning the years 2004 to 2015. The dataset was divided into training and validation sets at ratio of 7:3. Variables were screened by LASSO, and the Cox proportional hazards regression model was used to construct the 10-year risk prediction model of CVD. The model’s performance was assessed using the concordance index (C-index), receiver operating characteristic (ROC) curve, calibration plots, and decision curve analysis (DCA) for discrimination, calibration, and clinical utility. Results: This study included 5,186 individuals, with males accounting for 48.1% and a mean age of 46.39 ± 13.74 years, and females accounting for 51.9% and a mean age of 47.36 ± 13.29 years. The incidence density was 10.84/1000 person years. The model ultimately incorporates 11 non-invasive predictive factors, including dietary-related, demographic indicators, lifestyle behaviors, and disease history. Performance measures for this model were significant (AUC = 0.808 [(95%CI: 0.778–0.837], C-index = 0.797 [0.765–0.829]). After applying the model to internal validation cohorts, the AUC and C-index were 0.799 (0.749–0.838), and 0.788 (0.737–0.838), respectively. The calibration and DCA curves showed that the non-invasive model has relatively high stability, with a good net return. Conclusions: We developed a simple and rapid non-invasive model predictive of CVD for the next 10 years among Chinese adults.

## 1. Introduction

Cardiovascular disease (CVD) which includes a range of chronic conditions that impair heart and blood vessel function, is the main cause of global death [1], with nearly one-third of all deaths worldwide attributed to CVD [2]. China is a country with a heavy burden of CVD [3]. It has been reported that 330 million people suffer from CVD and two out of every five people die from CVD in China [4]. Identifying CVD’s risk factors to prevent it is critically important [5].

Many scientists have been exploring models for early prediction of CVD. Well-known examples include the Framingham [6] general CVD equations, Pooled Cohort Equations (PCE) [7] in the United States (US), the QRISK in the United Kingdom [8], the Systematic Coronary Risk Evaluation (SCORE) [9] model in Europe and the WHO cardiovascular disease risk prediction chart [10].The Framingham and PCE models require recalibration and updating before they can be effectively implemented in Asian populations [11]. The World Health Organization (WHO) CVD risk prediction chart has been found to underestimate the 10-year CVD risk in most regions of China [12]. Those models are based on Western samples, which limits their applicability to Chinese [11,13,14]. China’s CVD prediction model, known as China-PAR [15], encompasses variables that necessitate laboratory tests, such as comprehensive metabolic panel indicators, making them unsuitable for individuals to conduct real-time self-assessment of cardiovascular risk.

Poor dietary habits account for about 50% of cardiovascular deaths each year [16]. The important guidelines for CVD prevention have always recommended this controllable lifestyle for self-regulation [17,18]. It is necessary to incorporate a reasonable diet into the prevention of CVD [19]. Non-invasive predictive models incorporating dietary information are rarely reported. Therefore, we have utilized data from a 10-year follow-up obtained from the “China Health and Nutrition Survey (CHNS)” database to identify particular indicators to develop and confirm a straightforward, practical, and accurate tool for predicting the risk of CVD.

## 2. Methods

### 2.1. Study Design and Subject Selection

**Figure 1:**
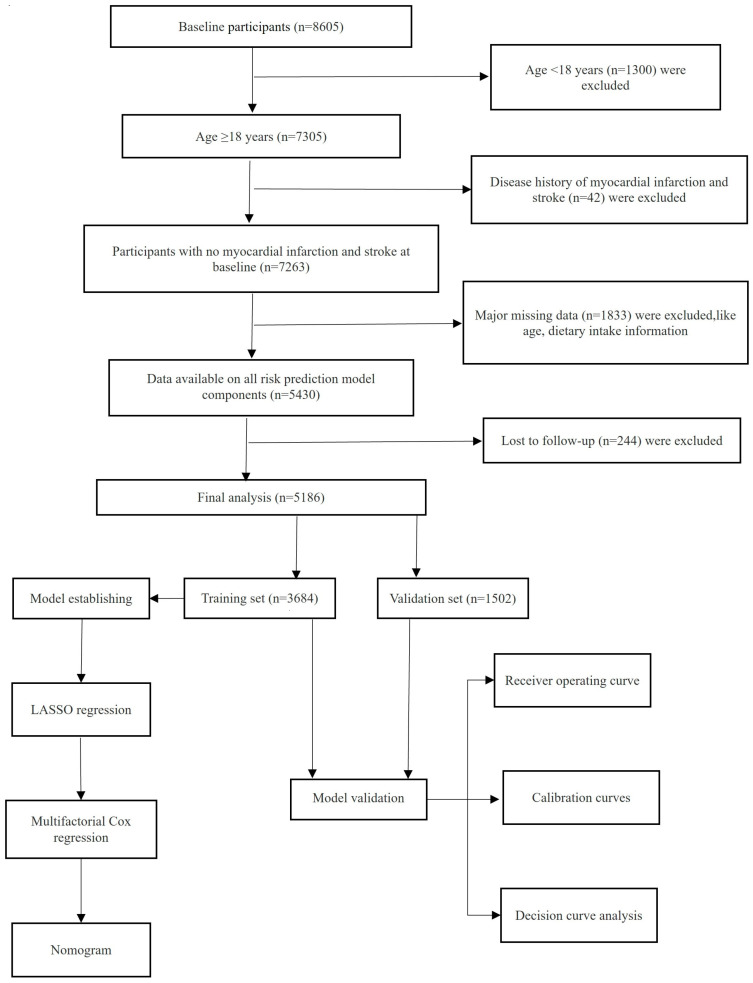
The established CVD prediction model and a flow chart for the selection of research subjects.

The research data utilized in this analysis were obtained from CHNS. This survey was designed to investigate the linkages between significant determinants such as socioeconomic status, dietary habits, levels of physical exercise, and overall health at various stages of life. To date, ten comprehensive survey rounds have been conducted, spanning the years 1989 to 2015. The survey covered nine provinces and used a multi-stage, random clustering process to draw a sample of each province. Written consent was obtained from all individuals participating in the CHNS study. The research received clearance from the ethics committees at the University of North Carolina at Chapel Hill and the National Institute of Nutrition and Food Safety, China Center for Disease Control and Prevention. The baseline data for this study were from August 2004 to December 2004, and the follow-up deadline was December 2015. Excluding residents younger than 18 years of age, patients with CVD at baseline, incomplete baseline data, lost to follow-up, and incomplete follow-up information left a total of 5186 individuals being incorporated into the final study group (Figure 1).

### 2.2. Data Collection

Standardized surveys were utilized to gather data on demographics, lifestyle information, and medical history. A person is considered a current smoker if they have smoked at least one cigarette daily for a year or longer. Alcohol consumption is defined as the average consumption of at least 50 g of alcohol per day for 1 year or more. A family history of atherosclerotic CVD is defined as at least one parent or sibling with myocardial infarction or stroke. Body mass index (BMI) is calculated by dividing a person’s weight in kilograms by their height in meters squared, with measurements taken in light indoor attire and without shoes.

### 2.3. Diagnostic Criteria

The primary outcomes of our study were myocardial infarction and all types of stroke. Myocardial infarction and stroke are the conclusions of a medical professional who diagnoses the study participants, and investigators record them through face-to-face or telephone interviews, such as asking the study participants, Has the doctor diagnosed you with myocardial infarction? Has the doctor diagnosed you with a stroke?

### 2.4. Predictive Variable Selection

We selected 17 variables based on a comprehensive literature review and clinical expertise. Structured questionnaires were used to collect information on sociodemographic factors and lifestyles, including age, sex (male, female), urbanization (urban, rural), marriage (unmarried, married, divorced and widowed), education (less than high school, high school or above), smoker (no, yes), alcohol (no, yes), hypertension (no, yes), antihypertensive therapy (no, yes), diabetes mellitus (no, yes), family history of CVD (no, yes) and BMI (lean and normal, overweight, obesity). BMI data were divided into three different groups: lean and normal weight group (<24 kg/m^2^), overweight group (24–28 kg/m^2^), and obese group (≥28 kg/m^2^). We assessed participants’ dietary intake through three consecutive 24-h dietary recalls and utilized the Chinese Food Composition Table (FCT) to convert the data into intake values for macronutrients including average daily kcal intake, average daily carbohydrate intake, average daily fat intake, and average daily protein intake. The geographic area was classified into northern (i.e., Liaoning, Heilongjiang, Shandong, and Henan) and southern regions (i.e., Hubei, Hunan, Guangxi, Guizhou, Jiangsu) based on the geographical demarcation of the Qinling-Huaihe line in China.

### 2.5. Statistical Analysis

A 7:3 ratio was applied to randomly assign participants to the training and validation sets. Missing information was filled by multiple interpolations, and the number of interpolations was 5. R software (Version 4.3.1) and SPSS software (Version 25.0) were employed for carrying out the statistical analysis. The two-sided test was used for statistical inference, and the *p*-value less than 0.05 was statistically considered significant. Continuous data were presented as mean with standard deviation, while categorical data were shown as proportions. Differences in continuous and categorical data were assessed using the Student’s *t*-test and chi-square test, respectively. The variables in the training set were screened by LASSO regression, and the variables with non-zero coefficients in the LASSO regression model were selected as candidate variables. Then, with the help of Cox proportional hazards regression, the risk prediction model of CVD was constructed, and finally the model with the smallest Akaike information criterion value was selected as the prediction model. Then we created a nomogram, to graphically represent predictions. The reliability of the risk prediction tool was gauged through multiple indicators, encompassing the ROC, C-Index, calibration charts, and DCA analysis. The AUC of an ROC curve ranges from 0.5 to 1, with a value near 1 signifying a model’s strong predictive performance. The C-index, representing the concordance rate between predicted and actual outcomes across all sample pairs, is a measure of a model’s discrimination capability, an index above 0.7 suggests high discriminative ability. A calibration plot, which scatter-plots actual versus predicted incidences, shows model accuracy when the plotted points align closely with the diagonal line of the coordinate system. DCA curves that surpass the boundaries of the highest and lowest values suggest that the model possesses good potential for practical use in clinical settings.

## 3. Results

### 3.1. General Characteristics of Training and Validation Sets

This study enrolled 5186 adults, having a mean age of 46.89 ± 13.51 years, with 2496 (48.1%) being male and 2690 (51.9%) being female. The average follow-up was 9.81 ± 2.23 years, and there were 276 new cases of CVD, with an incidence density of 10.84/1000 person-years. The entire group of participants in the study were segregated into two subsets: one for training, comprising 3684 individuals, and another for internal validation, with 1502 individuals. Training and validation sets did not exhibit a statistically significant difference except for geographical area (as shown in Table 1).

### 3.2. Construction of Prediction Models

**Figure 2:**
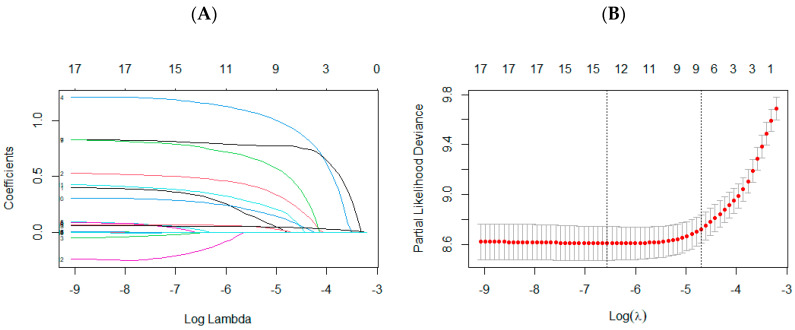
LASSO regression model was used to select factors for CVD. (**A**) The 17 colored lines in the figure represent 17 variables, which were screened using LASSO regression. The *x*-axis represents the log lambda, while the *y*-axis denotes the partial regression coefficient. As the logarithm of lambda rises, the regularization parameter strengthens, diminishing the bias regression coefficient’s magnitude, and potentially reducing it to near zero, leading to its exclusion. (**B**) The dashed line on the left is λ min, which means λ when the deviation is the smallest, which means that the model fitting effect is the highest under the lambda value. The 14 variables corresponding to log (λ) min were used.

**Figure 3:**
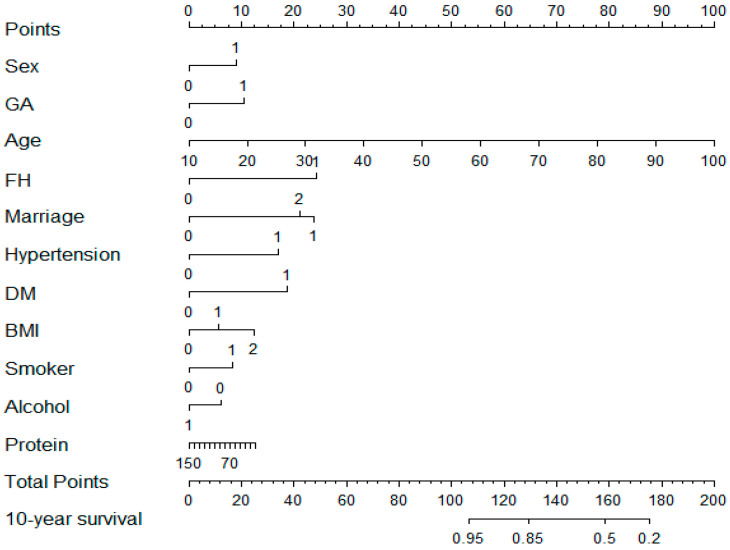
A predictive tool for the likelihood of CVD progression, the nomogram assigns points to each factor according to its scoring system. The aggregate of these points yields a total score, which, when aligned with the scale, provides an estimated chance of surviving CVD. GA, Geographic area (0 = South, 1 = North); FH, Family history of CVD (0 = No, 1 = Yes); Marriage, (0 = No, 1 = Yes); Hypertension, (0 = No, 1 = Yes); Diabetes mellitus, (0 = No, 1 = Yes); BMI, (0: <24.0, 1: 24.0~28.0, 2: >28.0); Smoker, (0 = Unmarried, 1 = Married, 2 = Divorced or widowed); Alcohol, (0 = No, 1 = Yes); Protein, average daily protein intake.

LASSO regression was used to identify key variables from 17 factors in the training set. As λ increased, the coefficients of less influential variables on CVD morbidity risk approached zero sooner, and there was a progressive reduction in the count of variables (Figure 2A). All predictors were screened out when the minimum MSE was increased (λ = 0.0007). A total of 14 variables were screened out, which were average daily carbohydrate intake, average daily protein intake, age, sex, urbanization, family history of CVD, marriage, hypertension, antihypertensive therapy, diabetes, BMI, geographic area, smoke and drink. Fourteen variables were included and subjected to the multivariate Cox proportional hazards regression analysis (Table 2). The variables retained after further screening by the stepwise regression method were age, sex, urbanization, family history of CVD, marriage, geographic area, BMI, diabetes, smoke, drink, and average daily protein intake. Finally, these 11 variables were included in the nomogram (Figure 3). For example, in our nomogram, for a 60-year-old man from Sichuan, married, no family history of CVD, high blood pressure, no diabetes, smoking, and drinking, BMI is overweight, average daily protein intake is 70 g, the total score is 132, and the 10-year CVD survival rate is about 85%.

### 3.3. Validation of Risk Prediction Models

**Figure 4:**
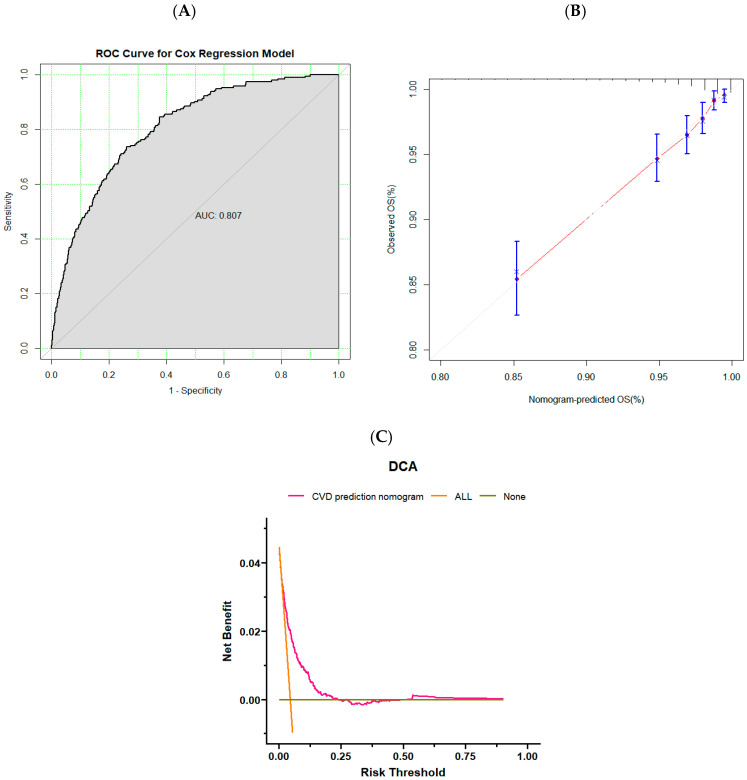
(**A**) ROC of the training set. (**B**) Calibration curves of the training set. Calibration curves illustrate the relationship between predicted and actual CVD risks. Risk predictions made by the model are depicted on the *x*-axis, in contrast to the actual risks displayed on the *y*-axis. The dashed line indicates perfect predictions by an ideal model, while the solid line represents the nomogram’s performance, with closer alignment to the dashed line indicating better accuracy. (**C**) DCA of the training set. The black line indicates the net benefit assuming no participants develop CVD, whereas the light orange curve signifies the net benefit assuming all participants are at risk of developing CVD. The clinical utility of the model is illustrated by the area between the pink curve and the light orange curve.

**Figure 5:**
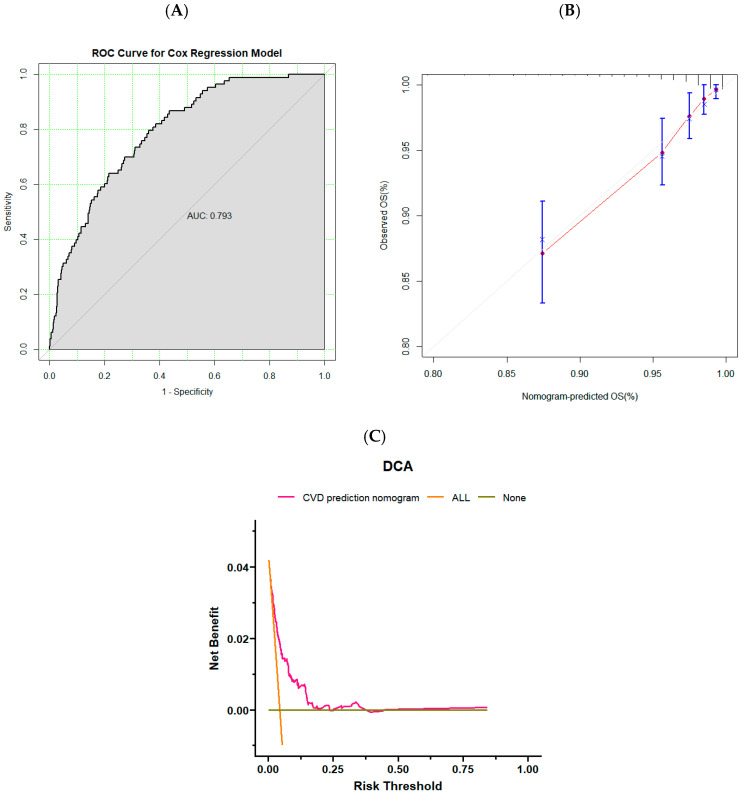
(**A**) ROC of the validation set. (**B**) Calibration curves of the validation set. (**C**) DCA of the validation set.

We utilize training and validation sets to evaluate established models for predicting CVD in adults. The C-index of the model was 0.797 (0.765–0.829) in the training set and 0.788 (0.737–0.838) in the validation set. The AUC was 0.808 (0.778–0.837) in the training set and 0.799 (0.749–0.838) in the validation set (Figure 4A and Figure 5A), both of which showed that the prediction model had good prediction performance. The training and validation sets were confirmed using calibration curves (Figure 4B and Figure 5B). The calibration curves were close to the dotted line in the calibration chart. It proved that the predicted survival rate of cardiovascular disease is close to the actual observed survival rate. Therefore, the prediction model has good calibration ability. Finally, the DCA of the training and validation sets (Figure 4C and Figure 5C) were analyzed, which were located in the range of 0.1–0.25 in horizontal coordinates, indicating that the benefit of using this model to predict CVD incidence was greater than that of patients receiving all treatment or no treatment if the threshold for the incidence probability of a patient was 10–25%. In the range of 10–25%, the net benefits are available.

## 4. Discussion

The present study screened variables through LASSO and constructed a 10-year risk prediction model for CVD using a Cox proportional hazards regression model. The performance of the model training and validation sets was evaluated using the C-index, ROC curve, calibration chart, and DCA, and it was found that the training set had good results in discrimination, calibration, and clinical utility. The validation set also yielded the same result. Historically, models for predicting CVD have used laboratory indicators, or not included the intake of macronutrients in people’s daily lives [6,7,8,9,10,15]. In this research, our ultimate findings reveal that the average daily protein intake in dietary macronutrients is associated with CVD. So, we tried to create a non-invasive CVD prediction model incorporating dietary information.

Firstly, we found that models without blood biochemistry can also predict CVD well. Secondly, lipid acquisition is more difficult and expensive than blood pressure, and test reports for laboratory indicators take time to obtain. Finally, some community residents are reluctant to draw blood, and in the case of limited medical resources, a model that does not rely on lipid information will be advantageous [9,10,20,21]. In contrast, most previous cardiovascular prediction models require laboratory indicators [6,7,8,9,15], which is not convenient for the early management of individual CVD. As a result, none of the 11 predictors in our study included blood biochemistry markers. We used a non-invasive model to predict CVD. Non-invasive predictive models are also involved in areas such as migraine and Parkinson’s disease [22,23]

Second, in addition to the metrics already included in previous models [6,7,8,9,10,15] such as age, sex, diabetes mellitus, smoker, and marriage [24], hypertension [25,26], alcohol [27,28], BMI [29], geographic area [30], and family history of CVD [31,32] are also associated with CVD, and our prediction model further uncovered the relationship between average daily protein intake in dietary macronutrients and CVD. An unhealthy diet significantly contributes to the risk of CVD [33]. Macronutrients constitute the primary elements of the human dietary intake [34]. However, the non-laboratory model developed by the WHO does not include dietary macronutrients [10]. A previous prediction model [20] with CVD was performed using the China Kadoorie Biobank and included fruit, meat, and eggs but not overall macronutrient intake. In our prediction model, average daily protein intake is a significant independent predictor [35]. Epidemiological studies indicate a strong correlation between higher protein consumption and a reduced likelihood of CVD [36,37]. Lyman [37] scholars noted that dietary proteins serve various roles beyond protein synthesis, significantly contributing to satiety, cellular communication, and the regulation of body temperature and blood sugar levels, with these metabolic effects being particularly noticeable when consumption exceeds the recommended dietary intake. A study conducted by Richter et al. [38] emphasized that more protein-rich plant foods should be consumed, and some animal protein sources, such as seafood, eggs, low-fat dairy products, poultry, and lean meats, should be consumed in moderation to replace refined carbohydrates and processed meats, so as to reduce the risk of CVD.

Different models have different values for CVD. We followed the 5.0% and 10.0% CVD risk values of the China-PAR model as cut-off points [21]. In the nomogram, an individual at high risk of CVD can be considered if the risk of CVD is ≥10.0% at 10 years. High-risk individuals require immediate and intensive interventions to correct harmful habits like smoking, weight issues and poor diet, supported by medical supervision and possibly advanced imaging for cardiovascular risk assessment. A risk of disease of 5.0–9.9%, can be regarded as an intermediate-risk individual. Intermediate-risk individuals need to modify unhealthy behaviors, potentially with clinical guidance. Low-risk individuals have a risk of disease of less than 5.0% and should follow health guidelines to sustain their status. Risk thresholds do not solely dictate medical intervention; lifestyle changes are vital at all levels. Stratification helps pinpoint the high-risk individuals for precise and timely intervention, ensuring resources are used wisely for maximum prevention effectiveness.

This study possesses several notable strengths. Firstly, our study included protein intake in daily dietary macronutrients to make predictions, which differed from the factors included in previous prediction models. Secondly, the practicability of our model is an advantage, as the model does not use blood biochemical indicators such as blood lipids, which can reduce medical costs and is more conducive to the detection and intervention of CVD at the grassroots level and individuals.Nonetheless, there are biases in the collection of outcome variables, as well as potential changes in social demographics, dietary habits, and risk factors over the years. In addition, we did not conduct external validation or make short-term predictions, which requires further compensation from subsequent research.

## 5. Conclusions

We have developed a non-invasive model that incorporates dietary macronutrients to predict CVD in adults. This model applies to primary community health services and helps adults predict their CVD risk in a personalized way, and people can actively manage their health based on the predictions.

## Figures and Tables

**Table 1:** Baseline characteristics of training and validation sets.

	Total (n = 5186)	Validation Set (n = 1502)	Training Set (n = 3684)	*p*-Value
Follow-up time, y	9.81 ± 2.23	9.84 ± 2.20	9.81 ± 2,24	0.648
Age, y	46.89 ± 13.51	46.65 ± 13.50	46.99 ± 13.52	0.412
Sex, n (%)				0.524
Male	2496 (48.1)	712 (47.4)	1784 (48.4)	
Female	2690 (51.9)	790 (52.6)	790 (52.6)	
Geographic area, n (%)				0.030
North	2122 (40.9)	650 (43.3)	1472 (40.0)	
South	3064 (59.1)	852 (56.7)	2212 (60.0)	
Urbanization, n (%)				0.198
Urban	1427 (27.5)	394 (26.2)	1033 (28.0)	
Rural	3759 (72.5)	1108 (73.8)	2651 (72.0)	
Marriage, n (%)				0.422
Unmarried	351 (6.8)	91 (6.1)	260 (7.1)	
Married	4518 (87.1)	1320 (87.9)	3198 (86.8)	
Divorced or widowed	317 (6.1)	91 (6.1)	226 (6.1)	
Education, n (%)				0.119
Less than high school	2367 (45.6)	678 (45.1)	1689 (45.9)	
High school or above	2819 (54.4)	824 (54.9)	1995 (54.1)	
Family history of CVD, n (%)				0.301
Yes	319 (6.2)	101 (6.7)	218 (5.9)	
No	4867 (93.8)	1401 (93.3)	3466 (94.1)	
Hypertension, n (%)				0.262
Yes	362 (7.0)	95 (6.3)	267 (7.2)	
No	4824 (93.0)	1407 (93.7)	3417 (92.8)	
Antihypertensive therapy, n (%)				0.319
Yes	243 (4.7)	63 (4.2)	180 (4.9)	
No	4943 (95.3)	1439 (95.8)	3504 (95.1)	
Diabetes mellitus, n (%)				0.774
Yes	47 (0.9)	15 (1.0)	32 (0.9)	
No	5139 (99.1)	1487 (99.0)	3652 (99.1)	
BMI, n (%)				0.298
<24.0	3362 (64.8)	998 (66.4)	2364 (64.2)	
24.0 to <28.0	1414 (27.3)	391 (26.0)	1023 (27.8)	
≥28	410 (7.9)	113 (7.5)	297 (8.1)	
Smoker, n (%)				0.905
Yes	1739 (33.5)	506 (33.7)	1233 (33.5)	
No	3447 (66.5)	996 (66.3)	2451 (66.5)	
Alcohol, n (%)				0.743
Yes	1749 (33.5)	501 (33.4)	1248 (33.9)	
No	3437 (66.5)	1001 (66.6)	2436 (66.1)	
Averagedaily kcal intake, kcal	2238.69 ± 640.43	2214.31 ± 636.71	2248.63 ± 641.76	0.080
Average daily carbohydrate intake, g	332.27 ± 105.57	328.86 ± 103.79	333.66 ± 106.28	0.137
Average daily fat intake, g	67.94 ± 35.20	67.07 ± 35.94	68.30 ± 34.89	0.256
Average daily protein intake, g	66.95 ± 22.68	66.23 ± 22.51	67.24 ± 34.89	0.145

**Table 2:** LASSO and multivariate Cox regression analysis of cardiovascular predictors in the study population.

Characteristics	LASSO Regression		Multifactorial Cox Regression Analysis		
	Coefficients	λ. 1 min	Hazard Ratio	95% CI	*p*-Value
Sex	0.3482311288	0.0007369600	1.577	1.060, 2.344	<0.05
Age	0.0559861979		1.057	1.044, 1.071	<0.05
Geographic area	0.4996928955		1.697	1.258, 2.290	<0.05
Urbanization	−0.0359183200		-	-	-
Family history of CVD	1.1926354994		3.369	2.322, 4.887	<0.05
Marriage	0.0610897230		-	-	-
Unmarried	-		-	-	-
Married	-		3.278	0.803, 13.388	0.098
Divorced or widowed	-		2.883	0.649, 12.801	0.164
Hypertension	0.8170091153		2.353	1.662, 3.333	<0.05
Antihypertensive	0.0660699743		-	-	-
Diabetes mellitus	0.7864943617		2.552	1.221, 5.333	<0.05
BMI	0.2907036033		-	-	-
<24.0	-		-	-	-
24.0 to <28.0	-		1.328	0.958, 1.839	0.088
≥28	-		1.856	1.198, 2.875	<0.05
Smoker	0.3947906943		1.512	1.048, 2.181	<0.05
Alcohol	−0.2245586523		0.732	0.515, 1.039	<0.05
Average daily carbohydrate intake	0.0007983668		-	-	-
Average daily protein intake	−0.0060387404		0.995	0.9884, 1.002	0.162

## Data Availability

The raw datasets used in this study are accessible to the public on the following platforms: CHNS (http://www.cpc.unc.edu/projects/china, (accessed on August 2004 to December 2015)).
